# Persistent Transmission of Circulating Vaccine-Derived Poliovirus — Somalia, January 2017–March 2024

**DOI:** 10.15585/mmwr.mm7325a2

**Published:** 2024-06-27

**Authors:** Amalia Mendes, Gedi Abdi Mohamed, Mohamed Derow, Tasha Stehling-Ariza, Abdinoor Mohamed, Kumlachew Mengistu, Kelley Bullard, Irfan Elahi Akbar, Hemant Shukla, Mohammad Al Safadi, Maureen Martinez

**Affiliations:** ^1^Geospatial Research, Analysis, and Services Program, Agency for Toxic Substances and Disease Registry, CDC; ^2^World Health Organization, Kabul, Afghanistan; ^3^Somali National Institute of Health, Mogadishu, Somalia; ^4^Polio Eradication Department, World Health Organization, Geneva, Switzerland; ^5^Global Immunization Division, Global Health Center, CDC; ^6^Division of Viral Diseases, National Center for Immunization and Respiratory Diseases, CDC; ^7^Polio Eradication Department, World Health Organization, Amman, Jordan.

**Summary**

**What is already known about this topic?**

Circulating vaccine-derived polioviruses (cVDPVs), associated with oral polio vaccines, can cause paralysis among persons in areas with low population immunity to polioviruses. In Somalia, cVDPV type 2 (cVDPV2) transmission has been ongoing since 2017, highlighting considerable challenges in controlling transmission.

**What is added by this report?**

Since the 2016 global withdrawal of type 2 oral poliovirus vaccine, cVDPV2 has continued to circulate in Somalia, with 39 cases documented across multiple regions during 2017–2024. Despite extensive immunization activities, children in large portions of the country remain inaccessible, particularly in the South-Central region.

**What are the implications for public health practice?**

Focusing on innovative strategies to vaccinate children in inaccessible areas, addressing operational challenges, and ensuring quality immunization campaigns in accessible regions are critical to interrupting cVDPV2 transmission in Somalia.

## Abstract

Since the launch of the Global Polio Eradication Initiative in 1988, substantial progress has been made in the interruption of wild poliovirus (WPV) transmission worldwide: global eradication of WPV types 2 and 3 were certified in 2015 and 2019, respectively, and endemic transmission of WPV type 1 continues only in Afghanistan and Pakistan. After the synchronized global withdrawal of all serotype 2 oral poliovirus vaccines (OPVs) in 2016, widespread outbreaks of circulating vaccine-derived poliovirus type 2 (cVDPV2) have occurred, which are linked to areas with low population immunity to poliovirus. Officials in Somalia have detected ongoing cVDPV2 transmission since 2017. Polio vaccination coverage and surveillance data for Somalia were reviewed to assess this persistent transmission. During January 2017–March 2024, officials in Somalia detected 39 cVDPV2 cases in 14 of 20 regions, and transmission has spread to neighboring Ethiopia and Kenya. Since January 2021, 28 supplementary immunization activities (SIAs) targeting cVDPV2 were conducted in Somalia. Some parts of the country are security-compromised and inaccessible for vaccination campaigns. Among 1,921 children with nonpolio acute flaccid paralysis, 231 (12%) had not received OPV doses through routine immunization or SIAs, 95% of whom were from the South-Central region, and 60% of whom lived in inaccessible districts. Enhancing humanitarian negotiation measures in Somalia to enable vaccination of children in security-compromised areas and strengthening campaign quality in accessible areas will help interrupt cVDPV2 transmission.

## Introduction

Somalia has experienced decades of civil unrest since the 1991 fall of the central government, resulting in a complex, ongoing humanitarian emergency, population displacement, and health system collapse, especially in the South-Central region (*1*). Somalia’s administrative structure comprises 118 districts, 20 regions, and seven federal states, including the Banadir region (which includes the capital, Mogadishu); however, Somaliland operates autonomously in the north (*2*). As of December 2023, insurgents maintained partial or complete control in nearly one half of the 81 South-Central districts, obstructing house-to-house immunization activities for 17% of targeted children. Despite challenges, Somalia interrupted indigenous wild poliovirus (WPV) type 1 (WPV1) transmission in 2002 and stopped two WPV1 outbreaks resulting from importations during 2005–2007 and 2013–2014 (*2*,*3*).

In 2016, following the 2015 eradication of WPV type 2 (WPV2), a global, synchronized switch from the use of trivalent oral poliovirus vaccine (tOPV) (containing Sabin vaccine strain serotypes 1, 2, and 3) to bivalent OPV (bOPV) (containing types 1 and 3) was implemented to mitigate circulating vaccine-derived poliovirus (cVDPV) type 2 (cVDPV2) emergence risks (*3*,*4*). Emergence of cVDPVs (vaccine viruses that have reverted to neurovirulence) can occur after prolonged circulation of vaccine virus in underimmunized communities (*5*,*6*). Somalia’s immunization schedule includes 4 doses of bOPV and 2 doses of injectable inactivated poliovirus vaccine (IPV), which contains all three serotypes. Since 2017, national 3-dose OPV coverage in Somalia among infants aged ≤12 months through routine immunization has been estimated at 47%. National coverage with 1 IPV dose has been estimated at 42% since 2018 (*7*). Before the switch to bOPV, Somalia successfully interrupted prolonged (2008–2013) cVDPV2 transmission. In October 2017, Somalia detected cVDPV2 transmission in Banadir, followed by detection of cVDPV type 3 (cVDPV3) 4 months later. This report describes the ongoing activities and challenges in interrupting transmission of cVDPV2 in Somalia from 2017 to the present, highlighting progress and identifying areas requiring focused intervention.

## Methods

### Review of Somalia Polio Immunization Coverage and Vaccination Campaign Data

Surveillance and supplementary immunization activity (SIA)* data during January 2017–March 2024 were provided by the World Health Organization (WHO) Somalia Country Office (WHO Somalia). Routine 2017–2022 childhood immunization data (reported through June 26, 2023) are from WHO and UNICEF estimates of national immunization coverage reports (*7*). To determine SIA performance quality, administrative data and postcampaign surveys, including lot quality assurance sampling (LQAS)^†^ surveys (implemented in accessible areas only), were reviewed. Because a national census has not been conducted in >40 years, target population estimates are based on United Nations’ estimates. In 2023, WHO Somalia conducted a village accessibility survey, marking villages that were unreachable during SIAs because of insecurity as inaccessible (WHO Somalia data manager, personal communication, May 15, 2024).

### Review of Somalia Polio Surveillance

**Acute flaccid paralysis surveillance.** Acute flaccid paralysis (AFP) surveillance detects the recent onset of limb weakness among children. WHO performance indicator standards for AFP surveillance sufficiently sensitive to detect a case of polio in outbreak areas include the detection of three or more nonpolio AFP cases^§^ per 100,000 children aged <15 years per year and the collection of adequate stool specimens^¶^ from ≥80% of persons with AFP. During AFP case investigations, histories recalled by caretakers of the number of poliovirus vaccine doses received by the child are recorded.

**Environmental surveillance.** Environmental surveillance (ES) for poliovirus in Somalia is conducted via systematic sewage sampling at 17 sites in 11 administrative regions in six states. Genomic sequence analyses of the region coding the viral capsid protein (VP1) were reviewed to determine genetic relationships among polioviruses identified in ES samples and stool specimens from AFP patients. These activities were reviewed by CDC, deemed not research, and conducted consistent with applicable federal law and CDC policy.**

## Results

### Immunization Activities and Coverage

During the reporting period, 28 SIAs using OPV type 2 (OPV2) or IPV were conducted, including seven national immunization days, 12 subnational immunization days, and nine smaller, targeted campaigns.^††^ Twelve SIAs occurred in 2021, six in 2022, eight in 2023, and two in early 2024. Vaccines used included monovalent Sabin-strain OPV2 (mOPV2) (15 SIAs), novel OPV2 (nOPV2, further attenuated version of Sabin mOPV2 with enhanced genetic stability) (eight), tOPV (three), and IPV (two). During 2017–2020, 17 bOPV campaigns were conducted.

**FIGURE 1:**
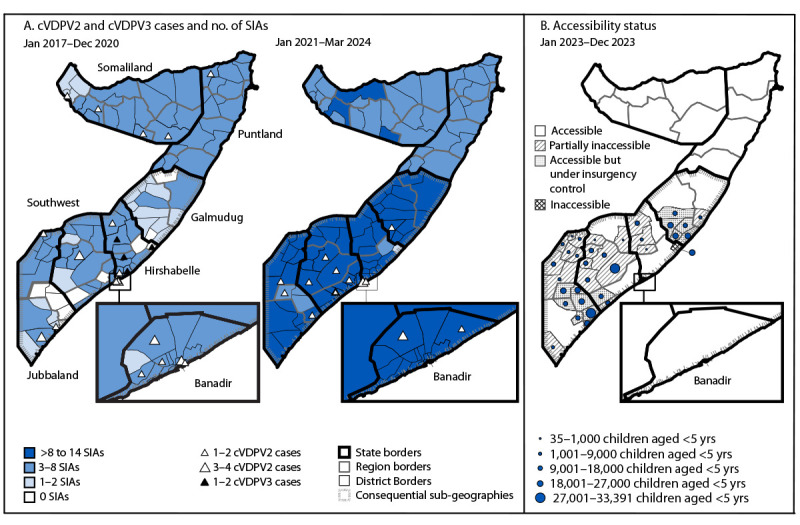
Reported cases* of polio caused by circulating vaccine-derived poliovirus type 2 and type 3, number of oral polio vaccine campaigns conducted, per district and period (A), and accessibility status (B)^†^ among children aged <5 years — Somalia, January 2017–March 2024^§^ **Abbreviations: **cVDPV = circulating vaccine-derived poliovirus; cVDPV2 = cVDPV type 2; cVDPV3 = cVDPV type 3; SIA = supplementary immunization activity; WHO = World Health Organization. * Total cVDPV2 cases by period: January 2017–March 2024 = 39; 2017–2020 = 23 (including one cVDPV2/cVDPV3 coinfection); January 2021–March 2024 = 16. ^†^ Children with poliovirus vaccine partially accessible are those from districts where parts of the district are under insurgency control, and no SIAs are conducted in those areas. Children with poliovirus vaccine accessible but under insurgency control are those from districts under complete control of insurgents but SIA operations are reportedly conducted with no verification or monitoring mechanisms in place. Children with poliovirus vaccine inaccessible are those from districts under full control of insurgents and no SIA operations are conducted. ^§^ Data as of May 16, 2024.

**Source:** WHO Geographic Information System Center for Health Administrative Boundaries, WHO Somalia population under 5-year estimates, accessibility status and reported cVDPV cases.

Among 1,921 children aged 6–59 months with nonpolio AFP reported during January 2017–March 2024, caretakers reported that 730 children (38%) had received ≥3 OPV doses through routine immunization, and that 538 (28%) had received ≥1 IPV dose; 1,364 (71%) children had received ≥3 OPV doses during SIAs. Overall, 231 (12%) children with nonpolio AFP were reported to have received no OPV doses through routine immunization or SIAs (i.e., zero-dose children); among these children, 219 (95%) were from districts in the South-Central region and 139 (60%) whose accessibility status was recorded were from inaccessible districts. As of December 2023, an estimated 472,743 children (representing 17% of children in Somalia aged <5 years) remain unreached for vaccination in the South-Central region because of security reasons (Figure 1). 

### AFP Surveillance

As of March 2024, the Somalia AFP surveillance system comprised 983 active surveillance sites and included a total of 796 village polio volunteers situated in all regions; 71% of these volunteers are in districts in the South-Central region. During 2017–2024, Somalia’s national nonpolio AFP rate consistently reached or exceeded three cases per 100,000 persons aged <15 years per year (annual range during 2021–2024 = 3.8–5.2). However, Banadir, the state reporting the highest number of cVDPV2 cases, consistently missed this target (range = 1.8–2.9) (Table).

**TABLE:** Acute flaccid paralysis surveillance performance indicators and administrative coverage, by state — Somalia, January 2021–March 2024*

Country/State	AFP surveillance performance indicators								Administrative coverage^†^ % vaccinated			
	2021		2022		2023		2024^§^					
	No. of AFP cases (% with adequate stool specimens^¶^)	NPAFP rate**	No. of AFP cases (% with adequate stool specimens^¶^)	NPAFP rate**	No. of AFP cases (% with adequate stool specimens^¶^)	NPAFP rate**	No. of AFP cases (% with adequate stool specimens^¶^)	NPAFP rate**	2021	2022	2023	2024
**Somalia**	**350 (93)**	**4.1**	**355 (96)**	**3.8**	**431 (92)**	**4.6**	**143 (88)**	**5.9**	**93**	**96**	**96**	**92**
Banadir	21 (95)	1.8	35 (97)	2.2	35 (80)	2.4	12 (92)	3.5	98	97	99	94
Galmudug	36 (92)	8.1	23 (100)	4.0	41 (93)	7.0	18 (72)	11.4	97	98	97	93
Hirshabelle	34 (94)	5.3	29 (97)	4.0	31 (94)	4.3	13 (85)	6.1	98	99	100	94
Jubbaland	60 (93)	5.2	50 (94)	4.2	69 (94)	5.9	29 (93)	9.3	92	94	93	91
Puntland	34 (97)	2.4	45 (100)	3.2	48 (96)	3.4	18 (94)	5.1	93	98	108	—^††^
Somaliland	109 (94)	6.7	103 (95)	5.9	124 (94)	6.9	36 (86)	7.8	92	91	89	—^††^
Southwest	56 (89)	2.8	70 (94)	3.2	83 (90)	3.7	17 (94)	3.0	93	96	95	91

**Abbreviations:** AFP = acute flaccid paralysis; NPAFP = nonpolio acute flaccid paralysis.

* Data as of May 16, 2024.

^†^ Proportion of targeted children aged <5 years who received a dose of poliovirus vaccine during a supplemental immunization activity.

^§^ NPAFP rate annualized from AFP surveillance data through March 2024.

^¶^ Adequate stool specimens are defined as two stool specimens of sufficient quality for laboratory analysis, collected ≥24 hours apart, both within 14 days of paralysis onset, and arriving in good condition at a World Health Organization–accredited laboratory with reverse cold chain maintained, without leakage or desiccation, and with proper documentation.

** Cases per 100,000 persons aged <15 years. The surveillance performance indicator target is three or more NPAFP cases per 100,000 persons aged <15 years per year. The NPAFP rate was not calculated for regions with <100,000 persons aged <15 years. https://polioeradication.org/wp-content/uploads/2020/02/Polio-surveillance-status-report-2019.pdf

^††^ No administrative coverage data reported for 2024 to date.

The 80% stool specimen adequacy target was met each year during 2017–2023 (annual range = 92.4%–99.1%) and in each state except Galmudug (72.2%) in the first quarter of 2024. However, the proportion of stool specimens that arrived at the WHO-accredited laboratory in Kenya within the recommended 3 days after collection decreased from 44% in 2017 to 9% in 2022 and 2023.

### Environmental Surveillance

During January 2017–March 2024, a total of 73 cVDPV2 isolates were detected across four of the six states with ES sites; Banadir state accounted for 66 (90%) detections. Collection sites in Somalia increased from four in 2017 to 17 in 2023.

### Epidemiology of cVDPV Cases

**FIGURE 2:**
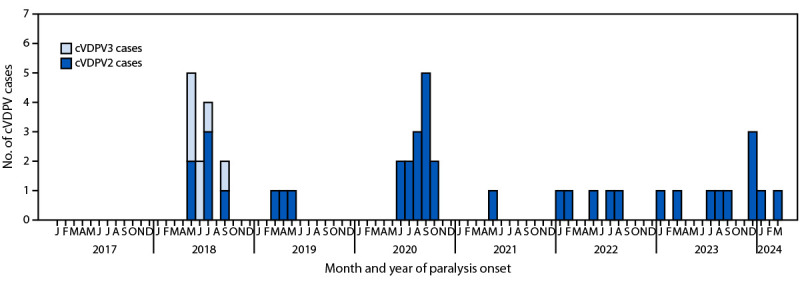
Number of circulating vaccine-derived poliovirus type 2 and type 3 cases, by month — Somalia, January 2017–March 2024*^,^^†^ **Abbreviations**: cVDPV2 = circulating vaccine-derived poliovirus type 2; cVDPV3 = circulating vaccine-derived poliovirus type 3; bOPV = bivalent oral poliovirus vaccine (OPV); mOPV2 = monovalent OPV type 2; NID = national immunization day; OPV = oral poliovirus vaccine; nOPV2 = novel OPV type 2; SNID = subnational immunization day; tOPV = trivalent OPV. * OPV campaigns were conducted as follows: three bOPV NIDs were conducted during 2017 (February, April, and December); three during 2018 (May, July, October, and November); two during 2019 (March and November); and three during 2020 (March, August, and December). Two mOPV2 NIDs were conducted during 2021 (June and July) and two in 2022 (March and June). Two nOPV2 NIDs were conducted during 2023 (August and November). One bOPV SNID was conducted during 2019 (April). One mOPV2 SNIDs was conducted during 2017 (December); four during 2018 (January, July, August, and September); four during 2019 (May, June, August, and September); two during 2020 (September and October); three in 2021 (February, March, and October); and three in 2022 (February, August, and October). One tOPV SNID was conducted during 2022 (November) and one in 2023 (February). Three nOPV2 SNIDs were conducted during 2023 (May, June, and July), and two during 2024 (February and March). ^†^ Data as of May 16, 2024.

**cVDPV2 cases.** During 2017–2024, 39 cVDPV2 cases were reported from all seven states, including 34 (87%) from districts in the South-Central region^§§^ (Figure 2) (Supplementary Table, https://stacks.cdc.gov/view/cdc/157501) (Village accessibility survey, WHO Somalia, unpublished data, 2024). The remaining five cases were reported from Somaliland (four) and Puntland (one) during 2019–2020. Overall, the mean age of patients was 36 months (range = 3–108 months) and 49% were female. Among the 39 cVDPV2 patients, 20 (51%) reportedly had received zero routine immunization or SIA OPV doses.

**cVDPV3 cases.** In 2018, states Hirshabelle and Jubbaland reported five and two cVDPV3 cases, respectively (Figure 2). Among these cases, three patients had received no routine immunization or SIA OPV doses, and four had received >3 SIA doses. No cVDPV3 has been isolated since September 2018.

### Genomic Sequence Analysis of cVDPV Isolates

In 2017, genomic sequence analysis of circulating cVDPV2 strains indicated protracted circulation of the SOM-BAN-1 cVDPV2 emergence occurred ≥3 years before detection. The most recent case in this emergence group was detected in Jubbaland in March 2024. During January 2021–March 2024, 17 (59%) of 29 isolates were detected in Banadir, including 10 of 16 orphan viruses,^¶¶^ which indicates substantial gaps in surveillance.

In December 2023, a second cVDPV2 emergence (SOM-BAY-1) was identified in southern Somalia and was most recently detected in January 2024. In addition, a cVDPV2 isolated from an ES sample collected in Banadir in May 2022 was from the Yemen cVDPV2 emergence group YEM-TAI-1; no further detections have occurred in Somalia to date.

## Discussion

Since 2017, 39 cVDPV2 cases have been detected in 14 of Somalia’s 20 regions. Many children in regions with ongoing poliovirus transmission have been inaccessible for more than a decade. An estimated 472,743 unreachable children in Somalia’s South-Central Region in the 2023 village-level inaccessibility survey represent approximately one in six (17%) children aged <5 years in Somalia. Along with chronically low routine immunization coverage, the quality of SIAs has also been compromised, even in accessible areas. Despite numerous SIAs responding to cVPDV2 transmission, caretakers of approximately one half of patients reported that the child had received no OPV doses.

Under new leadership, the Somalia polio team has undertaken several initiatives, including improving health worker training, expanding transit vaccination to reach mobile populations, enhancing SIA monitoring and postcampaign assessment, and working with humanitarian negotiators to gain temporary access to security-compromised areas. The Prime Minister of Somalia will lead a recently established Task Force on Polio Eradication and Immunization. The recent introduction of trained third-party monitors and enhanced postcampaign analysis under the new leadership in 2023 has led to a more realistic portrayal of LQAS outcomes. The decline in the percentage of districts passing LQAS assessments, from 87% in 2021 to 55% in November 2023, likely signals improved accuracy of SIA monitoring quality, indicating that inaccessibility is only one of multiple factors preventing children from being immunized. Comprehensively addressing SIA preparedness challenges and campaign implementation bottlenecks, strengthening surveillance and cross-border measures, and prioritizing humanitarian negotiation activities are critical to improving SIA implementation and interrupting cVDPV2 transmission.

### Limitations

The findings in this report are subject to at least five limitations. First, SIA data were incomplete for certain years. Second, uncertainty regarding inaccessibility limits accurate assessment of both surveillance and immunization activities. Third, receipt of OPV doses reported by caretakers might be subject to recall bias that could result in over- or underestimating coverage. Fourth, data on SIA OPV doses for persons with nonpolio AFP does not distinguish OPV2 from bOPV and might obscure specific patterns of immunity linked to each vaccine type. Finally, in the absence of a recent census, estimating accurate population data for target groups in Somalia remains challenging.

### Implications for Public Health Practice

Interrupting cVDPV transmission in Somalia is critical to keeping the Horn of Africa polio-free and interrupting cVDPV2 transmission worldwide. Strategies to achieve this goal include focusing on innovative approaches to vaccinate children in inaccessible areas, addressing operational challenges, and ensuring quality immunization campaigns in accessible regions.

## Footnotes

* SIAs are mass immunization campaigns intended to supplement the routine immunization systems and generally target children aged <5 years with OPV, regardless of previous vaccination history. In Somalia, SIAs are conducted primarily through house-to-house and site-to-site vaccination modalities.

† LQAS is a survey method to determine the quality of vaccination activities after SIAs in predefined areas (lots), such as health districts and involves dividing the population into lots to determine vaccination status. If the number of unvaccinated children in the sample exceeds three, the lot fails (i.e., at a pass threshold of ≥90%), and mop-up activities (i.e., search and vaccination of children recently missed) are recommended. If the 90% threshold is met, the lot is classified as having passed.

§ Nonpolio AFP cases are discarded as polio cases because they do not have laboratory or other evidence of poliovirus as the cause of the paralysis. The standard WHO performance indicator nonpolio AFP target for sufficiently sensitive surveillance to detect a case of polio for countries with outbreaks or endemic transmission is three or more cases per 100,000 children aged <15 years per year. https://polioeradication.org/wp-content/uploads/2020/02/Polio-surveillance-status-report-2019.pdf

¶ Adequate stool specimens are defined as two stool specimens of sufficient quality for laboratory analysis (i.e., arriving in good condition at a WHO-accredited laboratory with reverse cold chain maintained, without leakage or desiccation, and with proper documentation), collected ≥24 hours apart, both within 14 days of paralysis onset. The global standard surveillance performance indicator target is collection of adequate stool specimens from 80% or more of AFP cases.

** 45 C.F.R. part 46, 21 C.F.R. part 56; 42 U.S.C. Sect. 241(d); 5 U.S.C. Sect. 552a; 44 U.S.C. Sect. 3501 et seq.

†† Two fractional IPV campaigns (a dose-sparing strategy that uses one fifth of a standard IPV dose, administered intradermally), one measles-polio integrated campaign, one case-response campaign, and five short-interval additional dose OPV campaigns (<14 days between doses).

§§ Among 39 cVDPV2 cases reported during 2017–2024, 34 from the South-Central Region included those in the following districts: Banadir state: 12, South-West: nine (Bay [seven] and Lower Shabelle [two]), Jubbaland: eight (Gedo [two], Lower Juba [four], and Middle Juba [two]), Somaliland: four (Awdal [one], Galbeed [one], Sool [one], and Togdher [one]), Hirshabelle state: three (Hiran [two] and Middle Shabelle [one]), Galmudug: two (Galgadud), and Puntland: one (Bari).

¶¶ Orphan viruses are ≥1.5% divergent from their closest genetic match (i.e., ≤98.5% identity) and can indicate gaps in AFP surveillance based on the Global Polio Laboratory Network analyses of genetic divergence of the 900-nucleotide VP1 capsid protein coding region of poliovirus isolates.
